# Comment on “Pain in people living with HIV/AIDS: a systematic review (Parker *et al*. 2014)”

**DOI:** 10.7448/IAS.17.1.19096

**Published:** 2014-05-27

**Authors:** Richard Harding, Lorraine Sherr

**Affiliations:** 1 Global Research Programmes and Partnerships, Department of Palliative Care, Policy & Rehabilitation, Cicely Saunders Institute, King's College London, London, United Kingdom; 2 Clinical and Health Psychology, Department of Infection and Population Health, University College London, London, United Kingdom

Dear Editors,

We were pleased to see the systematic review of pain prevalence and management in the Journal of the International AIDS Society [1]. The clinical and research neglect of pain among people living with HIV is a cause of great concern [2], especially as evidence demonstrates that it can be effectively controlled [3]. There are a number of important reasons as to why pain among people living with HIV should be a central clinical and public health concern, an aspect that was not elaborated in the recent review. There is evidence that pain and symptom burden are associated first with sexual risk taking [4], second with poor adherence to antiretroviral therapy (ART) [5], third with treatment switching [6], fourth with viral rebound [7], fifth with poor quality of life [8] and sixth with suicidal ideation [9].

There are several reasons for this lack of attention to pain, including patients' belief that pain must be endured with an HIV diagnosis, physicians' lack of recognition of pain and their reluctance to enquire, document, monitor and treat pain symptoms [10]. Evidence suggests that HIV physicians detect only a third of patients' problems during clinical encounters [11,12]. In low- and middle-income countries, recent evidence has revealed poor availability of analgesics in pharmacies of HIV care facilities, not just with opioids but also with “step 1” analgesics such as paracetemol, with stockouts of analgesics being common [13].

The authors suggest that broader concepts of pain might be useful in this population. New evidence has identified the constituent components of self-reported pain, which reflect the physical, psychological and social dimensions of pain experienced by people living with HIV, especially in low- and middle-income countries [14].

We would also like to note that the search strategy employed by the authors in the March 2012 review fell short of retrieving and reporting some key data [1]. Specifically, data on pain among ART patients in South Africa found a seven-day period prevalence of 51.2% [15]; a study of newly diagnosed people in Uganda reported a seven-day period prevalence of 76% [16]; a study of HIV outpatients in Tanzania reported 41.4% point prevalence [17]; a UK community sample of men found a seven-day period prevalence of 42.6% [18], whereas a UK outpatient sample reported a seven-day period prevalence of 53.2% [19].

These data that were missed in the review go some way in addressing the focus of studies on high-income countries, and also a number of these additional papers address the issue of pain for those on ART, which has been a concern since the advent of new treatments [20]. Given the agreement across studies of high pain prevalence, we call for intervention studies to improve assessment and control of pain. We also call for studies to address the issue of pain among children, which was beyond the objectives of the present review, but has been noted as an area of great need but little evidence in a review of pediatric palliative care in sub-Saharan Africa [21].
